# Transparent conductor-embedding nanocones for selective emitters: optical and electrical improvements of Si solar cells

**DOI:** 10.1038/srep09256

**Published:** 2015-03-19

**Authors:** Joondong Kim, Ju-Hyung Yun, Hyunyub Kim, Yunae Cho, Hyeong-Ho Park, M. Melvin David Kumar, Junsin Yi, Wayne A. Anderson, Dong-Wook Kim

**Affiliations:** 1 Department of Electrical Engineering, Incheon National University, Incheon 406772, Korea; 2 College of Information and Communication Engineering, Sungkyunkwan University, Suwon 440746, Korea; 3 Department of Physics, Ewha Womans University, Seoul 120750, Korea; 4 Applied Device and Material Lab., Device Technology Division, Korea Advanced Nanofab Center (KANC), Suwon 443270, Korea; 5 Department of Electrical Engineering, University at Buffalo, State University of New York, Buffalo, New York 14260, USA

## Abstract

Periodical nanocone-arrays were employed in an emitter region for high efficient Si solar cells. Conventional wet-etching process was performed to form the nanocone-arrays for a large area, which spontaneously provides the graded doping features for a selective emitter. This enables to lower the electrical contact resistance and enhances the carrier collection due to the high electric field distribution through a nanocone. Optically, the convex-shaped nanocones efficiently reduce light-reflection and the incident light is effectively focused into Si via nanocone structure, resulting in an extremely improved the carrier collection performances. This nanocone-arrayed selective emitter simultaneously satisfies optical and electrical improvement. We report the record high efficiency of 16.3% for the periodically nanoscale patterned emitter Si solar cell.

Researchers have devoted to realize the high-performing photovoltaics (PVs)^1^,^2^,^3^,^4^,^5^ to substitute the traditional and strong dependency on fossil energy resources. Generally, we may think two approaches to realize the high-efficient solar cells by considering electrical and optical aspects^6^,^7^,^8^,^9^,^10^. One is to drive more light energy into a light absorber, which can be achieved by reducing the reflection at a surface^5^,^6^,^7^,^8^,^9^,^10^,^11^,^12^,^13^,^14^,^15^,^16^,^17^,^18^,^19^,^20^,^21^,^22^,^23^,^24^,^25^,^26^. The other approach is to minimize the electrical loss, which enables to collect more number of photo-generated carriers with a minimum pay for recombination loss^5^,^24^,^27^,^28^.

Unfortunately, these two important issues are not compatible so far. This is caused by an unbalanced design in which, more weightage is given to the optical aspects. Gigantic progresses for the improvement of optical enhancements have been achieved in PV technologies^6^,^18^,^29^,^30^,^31^,^32^. According to researchers' consistent efforts, various types of entities, such as nanowires, nanostructures and microstructures have been applied for PVs^5^,^6^,^7^,^8^,^12^,^13^,^14^,^15^,^16^,^17^,^18^,^19^,^20^,^21^,^22^,^23^,^24^,^25^,^26^. We already achieved the near zero reflector for broadband wavelengths^12^,^16^,^17^,^33^. Meanwhile, the practical performances of the fancy solar cells^5^,^8^,^12^,^14^,^15^,^16^,^17^,^18^,^19^,^20^,^21^,^22^,^23^,^24^ are still much behind from the readily-achievable commercials^3^,^31^. Moreover, the fabrication of conceptual nanoscale solar cells is usually limited in large-scale applications.

We conceived a functional nanoscale design to take advantages over both the optical and electrical benefits. The biggest question in front of the photovoltaic researchers is “Is it possible to minimize an inevitable electrical loss and meanwhile, to maximize the optical enhancement in a solar cell?” The periodically shaped nanoscale emitter could be the possible solution to realize such a device. Periodically nanoscale grating structures can efficiently improve the optical path lengths with the substantial reduction of light reflection, therefore these structures provide a strong means for the optically enhanced PVs^1^,^11^,^33^,^34^. For an electrical aspect, a heavily-doped emitter provides a low-resistive path and therefore adds an advantage for carrier transportation. However, the higher dopant concentration easily degrades a carrier lifetime due to the Auger recombination^6^,^35^, while lightly-doped emitter is effective to relieve the serious recombination concern. At the same time, the dopant concentration in lightly-doped emitter causes a high contact resistance. These two conflicts limit the design scheme of an emitter. A selective-emitter has emerged to acquire the advantages of both the lightly- and heavily-doped emitters by sectioning the emitter regions for doping levels^8^,^36^,^37^. Conventional methods for the selective-emitter were achieved by using laser diffusion and metallization^38^. These methods were developed for a planar structure and therefore limited to apply for the nanoscale entities.

We herein, propose and demonstrate the nanoscale patterned Si solar cell, which have periodical selective-emitter arrays. This design was achieved by nanoimprint method which is suitable for large-scale device fabrication. Commercial wet-etching process was applied to form the light-doped emitter region; meanwhile the heavily-doped emitter region was remained. As a result, we report the highest solar cell efficiency of 16.3% among the periodically nanoscale-patterned Si solar cells up to date. This result is substantially of improved efficiency compared to 13.7% of all-back-contact Si solar cells^5^, 13.8% of compound InP nanowires solar cells^21^,^22^ or our previous nanolens solar cells^1^.

In this paper the following challenges and topics are addressed:

- Large-scale fabrication of nanoscale Si patterns to have optical and electrical functions.
- Optical enhancements to suppress light reflection at Si surface.
- Electrical designs and performances of a selective-emitter solar cell.
- Electrostatic analysis for p/n junction and graded electric field distributions.
- Traces of light propagation profiles along the nanoscale patterns.

## Results

### Wet-etching without a mask

To tailor the surface of the Si substrates, a wet-etching method was applied. Si (100) wafer has a degree of anisotropy and thus etching rates are different to crystal planes due to the difference of the Si bonding energy. The (111) planes work as an etching barrier, while the (100) planes are easily etched away. We used the wet-etching solution with a mixture of NaOH (2.5 wt%), isopropyl alcohol (5 wt%), and de-ionized water (92.5 wt%).

**Figure 1:**
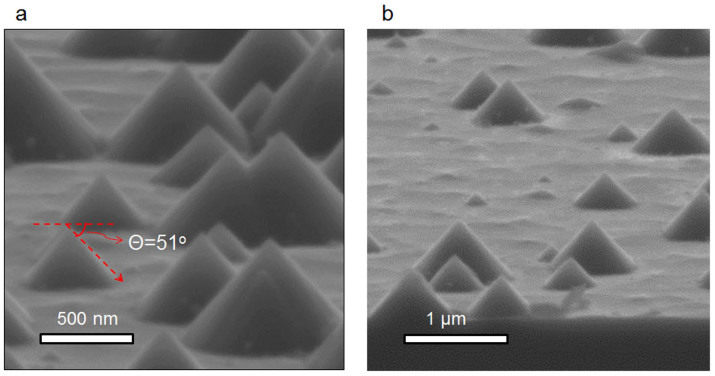
SEM observation for a textured Si sample. Direct wet-etching process formed (a) a uniform pyramidal angle of 51°. However, the sizes are varied by each pyramid (b).

Figure 1a shows a SEM image of the pyramidal shaped Si substrate, having an angle of about 51° between the (100) and (111) crystalline planes. The angle formation seems to be uniform through the pyramids, however, the sizes are varied by each pyramid (Fig. 1b) due to the different etching rate along the (100) plane even in a same solution^39^. Hereafter, this sample is referred as a textured Si sample.

### Wet-etching with a mask for periodically patterned nanocone arrays

**Figure 2:**
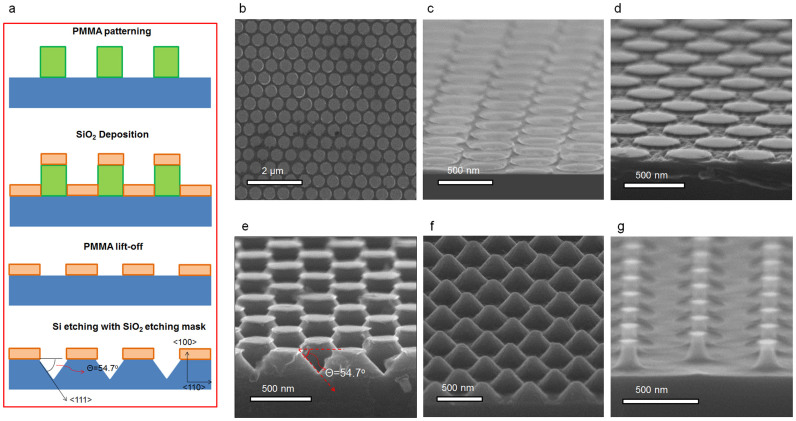
Fabrication of periodic nanoscale Si structures. (a) Process flows. (b) PMMA patterns. (c) SiO_2_ patterns for etching-masks. Observation images of Si etched for (d) 2 min, (e) 4 min, (f) 8 min, respectively. (g) A demonstration for over-etched Si structures.

In order to fabricate periodic nanoscale structures, a large-scale available nano-imprint method^40^ was applied to fabricate an etching mask. The fabrication sequences are presented in Figure 2a. A polymethyl methacrylate (PMMA) layer was patterned as hole-arrays on a Si substrate, as shown in Figure 2b. After then, a thin SiO_2_ (20 nm) layer was deposited on the hole-arrayed PMMA template. A lift-off process using an acetone solution was performed to remove the PMMA template and thus spontaneously removed the SiO_2_ layer, sitting on the PMMA layer. This procedure remained the inversely replicated SiO_2_ patterns on a Si substrate, as shown in Figure 2c. This SiO_2_ pattern works as an etching mask during the wet-etching process, which effectively relieves the different etching rate along the (100) planes, resulting in periodic nanoscale pyramids with uniform height.

We investigated to modulate the Si features by varying the time duration for the etching step. Si substrates showed grooved shapes (Fig. 2d) and nanoscale-pyramid arrays (Fig. 2e) for 2 min and 4 min etching time respectively. Due to the SiO_2_ etching mask, we can produce the periodic nanoscale pyramids, which has an angle of 54.7° between the (100) plane and the sidewall.

Sharpening geometry is very efficient to reduce the light reflection, and thus for the enhanced solar cell performances. In order to achieve this structure, the optimum etching time is found to be as 8 min. Uniformly patterned nanocones were fabricated to have a height of about 240 nm and a width of about 400 nm in a period of 500 nm, as shown in Figure 2f. Hereafter, this structure is referred as a nanocone Si sample and used for a nanocone solar cell. As increasing the etching time, the Si may be over-etched to lose the etching shape. Figure 2g is shown for an experimental demonstration for the over-etched Si structures.

### Reflectance profiles

Photo-responses of a solar cell are strongly dependent on the utilization of the incident light. This can be realized by the reduction of the incoming light reflection on a surface, which spontaneously drives more photons into the light-active semiconductor layer. Generally, we can realize the reduction of the incident light by means of two approaches. One way is to pattern the surface of the light-absorbing layer. The other approach is to adopt the graded refractive profiles. In order to achieve these two benefits, we designed a solar cell structure which is having a thin ITO film on the periodically patterned Si structures. For comparisons, three different types of Si structures were prepared as follows, a planar Si, a textured Si and a nanocone Si.

**Figure 3:**
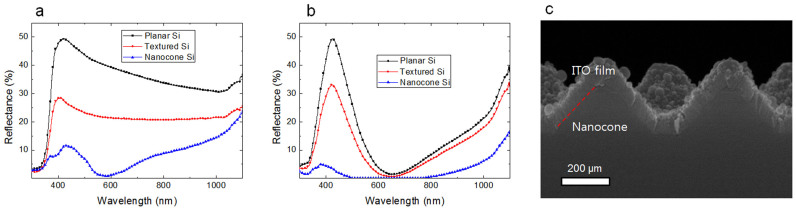
Reflectance profiles. (a) Reflectance profiles of a planar, a textured, and a nanocone Si substrate. (b) Reflectance profiles after coating an ITO film over a planar, a textured, and a nanocone Si substrate. (c) SEM image of 80 nm-thick ITO film coated nanocone Si arrays.

We clearly observed the structure dependent reflection tendencies from Figure 3a, showing the reflection profiles according to the Si structures. An average reflection value was obtained for the wavelength range of 300 nm–1100 nm. A planar Si gave a high reflectance value of 34.42%. Meanwhile, a textured Si effectively reduced the reflection value by 20.68%. A significantly reduced reflection value of 8.55% was achieved from the nanocone Si.

A thin ITO film was coated on the different Si substrate samples for further improvement. As shown in Figure 3b, all three samples obviously presented the AR coating layer effect by using an ITO coating on Si structures. For a planar Si substrate, the reflection value was reduced to be 16.99%. For a textured Si sample, the ITO coating significantly reduced the reflection value by 12.5%. Moreover, the ITO coating on the periodic nanocone Si surface substantially suppressed the reflection value by 2.68% and also provided the near-zero reflection for broad wavelengths. Less than 1% reflection range was spread out throughout the wavelengths between 472 nm and 826 nm in nanocone Si structure. This remarkably low reflection is attributed to the graded refractive effects of ITO-coating along with the conical shaped Si structures. Moreover, the electrically conductive ITO coating layer also contributes to reduce contact resistance^28^, which has been discussed in the later part.

Figure 3c is an SEM image of the Si nanocones with a top ITO layer. An ITO film actively works as an anti-reflection coating layer, due to its intermediate refractive index for the air-Si system. An optimum thickness of an ITO film was found to be 80 nm, which is calculated by the quarter wavelength method (*d = λ/4n*), in which *n* is the refractive index of the ITO^28^.

### Solar cell behaviors

**Figure 4:**
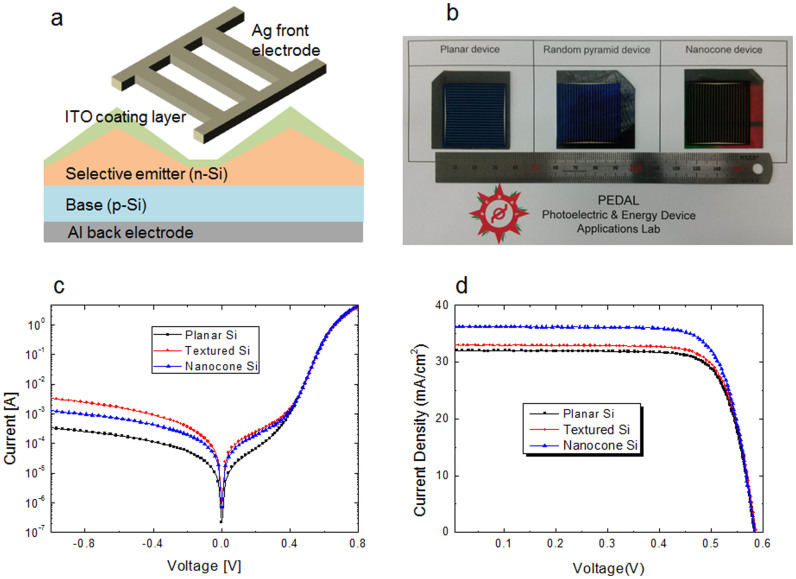
Solar cell performances. (a) Schematic illustration of a nanocone solar cell. (b) Photo images of solar cell samples. I–V characteristics under (c) dark condition and (d) one-sun illumination.

To fabricate solar cell, the front and back silver metal electrodes were applied. The schematics of a nanocone solar cell are presented in Figure 4a. Three different types of solar cells were prepared with a planar Si, a textured Si and a nanocone Si. Each device were tailored for a large size of 3.2 × 3.2 cm^2^, as shown in Figure 4b.

In order to investigate the diode characteristics, the I–V profiles for all devices were measured under dark condition. In general, all three samples provided good rectifying current flows (Fig. 4c). A planar Si device showed the lowest reverse saturation current (*I_RS_*), as expected. In comparison, a textured Si device and a nanocone Si device showed the increased I_RS_ values. The reverse current amount is directly related to the defects along the Si surface, which is caused by the etching process^20^,^24^,^25^,^26^. Surface defects increase the leakage current and is usually proportional to the surficial enhancement. It is noteworthy to mention here that the nanocone Si device has a lower *I_RS_* value than that of the textured Si device. This shows that the random-texturing induces the severe defect problem, due to the non-directional etching surface.

Under forward bias, we can observe the relation between the surface enhancement and increased current. At a positive 0.8 V, the nanocone device provided the highest forward current (I_F_) value of 4.12 A. Meanwhile, a random textured Si device showed a lower I_F_ value than that of a planar device. To overlook the dark current tendencies, the rectifying ratios which are calculated from ratio of the current value at +0.8 V to the current value at −0.8 V, were obtained and given in Table 1. A planar Si device has the highest rectifying ratio of 15152.5, due mainly to the lowest I_RS_ value. As next to the planar Si device, the nanocone Si device showed a rectifying ratio of 4367.3 which is higher than the 1499.5 of the random textured Si device.

In order to investigate a p-n junction quality, the diode ideality factor (*n*) was determined. The planar Si device provided *n* value of 1.52, showing a good p-n junction quality. Etching-processed devices also showed good *n* values of 1.57 for a nanocone device and 1.61 for a textured device. This implies that the recombination problem due to surface defects would be relieved by the surface enhancement. The *n* value is related to a rate function of the voltage change over the current change as shown in the following equation:
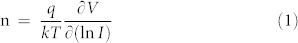
where kT and q are the thermal energy and electron charge, respectively. The surface enhancement for the nanocone device effectively improves the current value, and thus renders a fair *n* value.

In order to investigate the solar cell performances, the prepared devices were characterized using a simulator system (McScience-K3000) under one-sun (100 mW/cm^2^) illumination in connection with a power meter (McScience-K101). The periodic nanocone solar cell provided a substantially improved conversion efficiency of 16.3% (Supplementary section ‘Calculation of solar cell efficiency'), compared to 14.5% of a planar solar cell or 15.0% of a texture solar cell. This nanocone solar cell efficiency (16.3%) is considered to be the highest efficiency value among the periodic nanoscale-patterned Si solar cells up to date^22^. An open circuit voltage (*V_oc_*) is an important factor to accomplish a high efficient nanoscale solar cell. In general, the nanostructured solar cells tend to degrade *V_oc_* values by the trap density in a space charge region (*SCR*)^9^. From the results, all three samples have a relatively similar open-circuit voltage values (583–586 mV). There was no severe *V_oc_* degradation observed in the nanocone Si solar cell and this result is different from previous reports^15^,^17^,^23^. This result clearly demonstrates a fair formation of the *SCR* of the nanocone solar cell.

The enhanced efficiency of the nanocone solar cell is mostly attributed to the improved current value along with the periodic nanoscale Si feature. The short-circuit current density (*J_sc_*) of the nanocone solar cell was achieved to be 36.25 mA/cm^2^. This is a significantly improved current value from 32.05 mA/cm^2^ of the planar device. The current ratio (light-induced current/reverse saturation current) also directly controls the *V_oc_* value, according to the following relation:
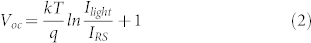
where *I_light_* is the light-induced current. For a nanocone solar cell, the defect-induced effect (increased *I_RS_* value) was sufficiently relieved by the significantly increased *I_light_*. Meanwhile, no significant current improvement was achieved for the texture solar cell (32.86 mA/cm^2^) due to the smaller surface than that of a nanocone Si device.

In order to investigate the current behaviors, the surface enhancements of the periodic nanocone structures have been calculated. Nanocones are periodically arrayed with a period of about 500 nm on the entire device surface. A single nanocone has a width of 400 nm and a height of 240 nm. By considering a planar surface (100%) as a reference, the nanocone structure has the surface area by 133.9% in a unit cell and thus increases the light-reactive Si region. This also simultaneously enlarges an interface between Si and ITO. Due to the increased ITO surface along the nanocone-arrays, the nanocone device provided a low series resistance value of 1.546 Ω cm^2^, which is much smaller than the 5 Ω cm^2^ of nanowire surface or close to a theoretical value^14^ of 1.5 Ω cm^2^.

In an electrical aspect, an ITO layer supports carrier transport from a Si to a metal electrode. As a result, the enhanced surface area by nanocone-arrays improved the light-induced current (*I_light_*) and diode forward current (*I_F_*), as well.

### Analyses for solar cells

**Figure 5:**
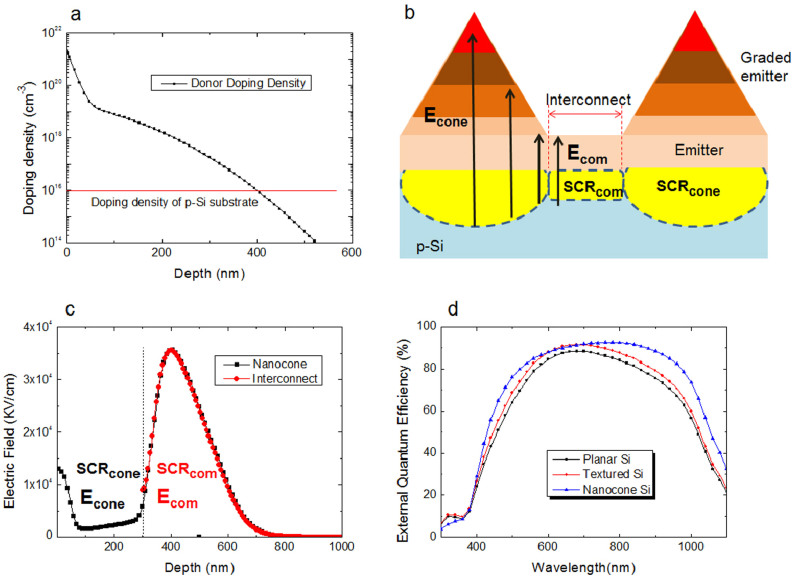
Selective emitter analyses. (a) Doping profiles for n-Si and p-Si. (b) Schematics of an emitter doping-graded nanocone solar cell. (c) Electric field distribution profiles. (d) Quantum efficiencies for solar cells.

A distinctive feature of the nanocone device is to have a selective-emitter. The etching process spontaneously tailors the emitter region to have convex nanocone-arrays, which is connected by an interconnect region. The interconnect region is located at a depth of 240 nm from the top surface. The doping profiles were analyzed using secondary ion mass spectroscopy investigation has been performed, as shown in Figure 5a. We obtained the donor concentration (*N_d_*) of about 10^18^/cm^3^ for the interconnect emitter region. Considering the acceptor concentration of the p-Si substrate (10^16^/cm^3^), the p/n junction between the n-Si emitter side and the p-Si base formed a space charge region (*SCR*). This is established by major carrier diffusion mechanism. Electrons move from n-Si to p-Si and holes move to the opposite direction. Electrons and holes, fixed in *SCR*, establish an electric field, which is a driving force to collect the photo-generated carriers.

A main advantage of the periodic nanocone device is to render the graded doping profile through each nanocone structure, as depicted in Figure 5b. The peak doping concentration (N_d_ = 10^21^/cm^3^) exists at a top of a nanocone and the doping concentration is gradually decreased with increasing distance from the top surface. This graded doping concentration in the nanocone geometry gives an additional electric field, and which correspondingly widens *SCR*.

The electrostatic profiles of the different shaped p/n junction were analyzed using PC1D program, as presented in Figure 5c. For the interconnect region, the electric field is distributed along the *SCR*, showing the peak value of 37 kV/cm at the center of *SCR*. This is caused by a common *E* (*E_com_*), co-existed both for a nanocone and an interconnect regions due to the common n-doping level (N_d_ = 10^18^/cm^3^). We denote the *SCR* formed by *E_com_* as *SCR_com_*, in Figure 5 (b, c). Different from the interconnect region, the nanocone-shaped emitter has the graded doping levels, which widens the *SCR* space, as denoted *SCR_cone_*. This was induced by an additional *E* distribution, which is denoted as *E_cone_*, in Figure 5c.

This doping-level graded emitter, having nanocone interconnected arrays, is spontaneously providing the feature of a selective emitter. The heavily-doped region contributes the carrier transport due to the enhanced *E*, which also provides a low-resistive path for photo-generated carriers. Meanwhile, the interconnect region has a benefit to reduce the recombination loss due to its low dopants concentration.

To examine the carrier collection performance, the external quantum efficiencies (EQE) were measured for all samples. The nanocone solar cell showed the remarkably enhanced carrier collection efficiencies for broad wavelengths compared to those of the texture or the planar devices, as shown in EQE (Fig. 5d). Precisely, the quantum efficiency of more than 85% is achieved by nanocone solar cell at a broad wavelength range of 600–900 nm which covers the important region for Si based solar cell devices. This clearly demonstrates that the nanocone-shaped selective emitter is very effective to collect the photo-generated carriers. It is worthy to note that QE improves enormously at longer wavelengths. At a wavelength of 1100 nm, the nanocone solar cell improved QE value by 50.6% from that of a planar solar cell (Supplementary section ‘Relative EQE). For a wavelength of 600 nm, a relatively low QE improvement (~4%) was obtained. This is caused by the high QE value (84.62%) of a planar device inherent from a low reflectance at the corresponding wavelength (Fig. 3b). The nanocone structure is also effective for short wavelengths. At a wavelength of 440 nm, the nanocone device showed a 29.3% enhanced QE value from that of a planar device. However, we observed the degraded QE performances for λ < 360 nm. This is attributed to the defects-induced recombination effect along the enhanced Si surface, resulting in the degraded QE values at very short wavelengths. We can also consider the Auger recombination effect for a heavily-doped emitter region (N_d_ > 10^20^/cm^3^)^6^,^35^. By considering photon absorption for λ < 400 nm, Si has a very short absorption length less than 100 nm. This causes a serious Auger recombination loss for the short wavelength photons. We can observe that the texture Si device showed the improved QE values at short wavelengths, due to its relatively light doping concentration in an emitter.

### Light behaviors by the nanocone structure

Besides the electrical features, it is also critical to investigate the light propagation of incident light behaviors through the nanocone geometry. To understand the optical characteristics of the nanocone solar cells, Maxwell's equations were numerically solved using the finite-difference time-domain (FDTD) simulations (Lumerical FDTD Solutions). The devices were modeled with unit cells and the proper boundary conditions. The light-induced electric field (*E_light_*) intensity distributions and reflectance spectra were obtained under illumination of linearly polarized light.

**Figure 6:**
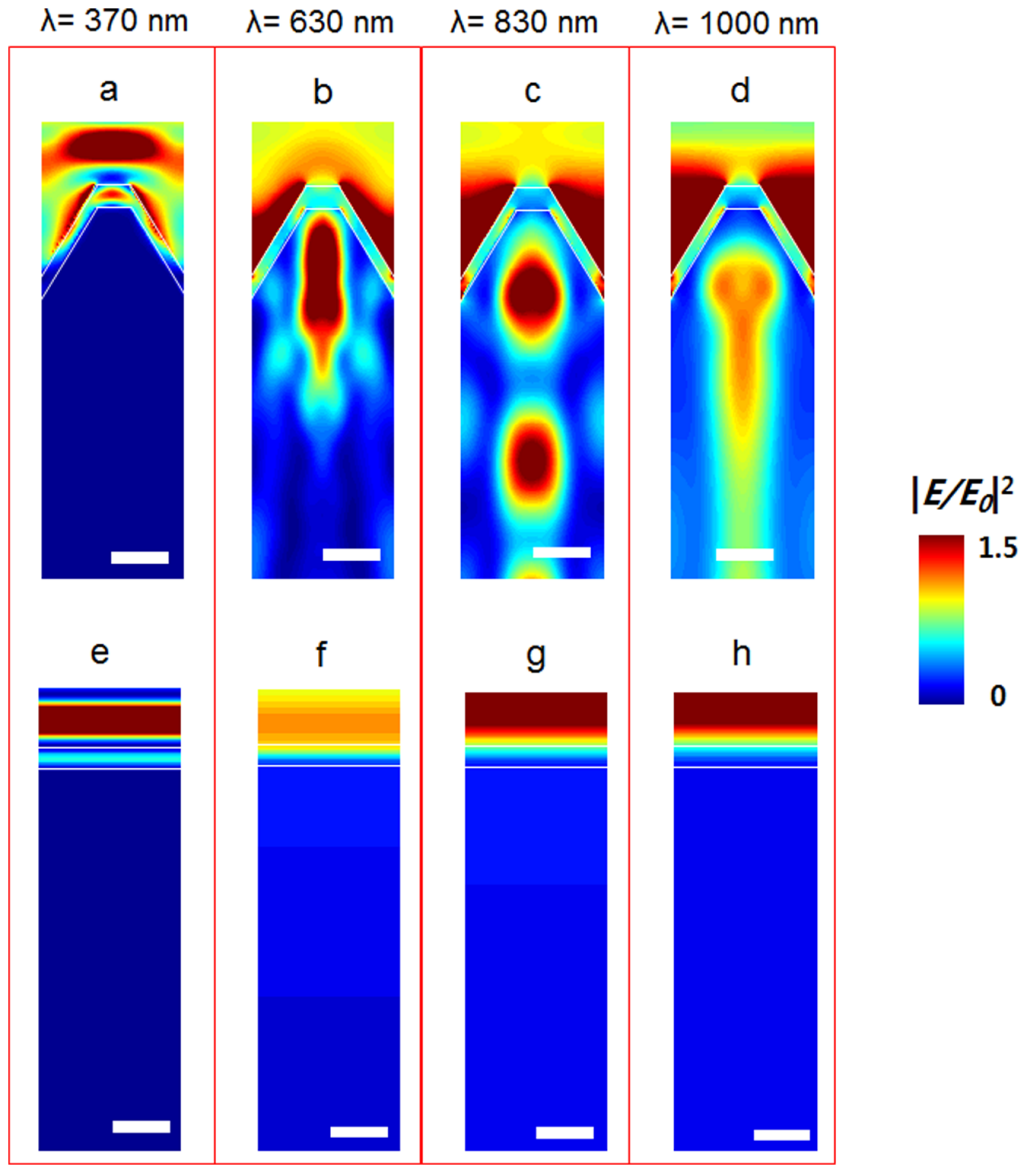
FDTD simulations. Light-induced electric field distributions for a different wavelength at 370 nm, 630 nm, 830 nm, and 1000 nm for a nanocone Si structure (a–d) and a planar Si (e–f). The scale bar is 100 nm.

Figure 6 shows the electric field distributions for a nanocone structure (a–d) and a planar structure (e–f), at different wavelengths (λ). For a planar structure, the incident light propagates as a form of plane wave. Meanwhile, the incoming light is significantly concentrated near the nanocone surface. The light propagation is modulated by passing the nanocone structure and *E_light_* distribution profiles are highly dependent on the light wavelengths.

At λ = 370 nm, the largest field intensity appears at the air/ITO interface and the intensity strongly decays in the ITO layer. As ITO layer is an electrical conductor, it contributes the photo-generated carrier collections. In contrast, the ITO film causes a free carrier loss, without creating the electron-hole pairs for short wavelengths^41^,^42^. This suggests that very limited incident photons can reach the Si active region in the solar cell, resulting in negligibly small QE at λ < 400 nm (Fig. 6a).

At λ = 630 nm, very strong light intensity is seen in the center of a nanocone region (Fig. 6b). Such field pattern could be attributed to the excitation of the resonant guided modes and the antireflection effects due to the nanocone patterns.

For long wavelengths (λ = 830 and 1000 nm), the nanocone structure generated strong *E_light_* and which propagated into a deep Si region, along the center line of a nanocone (Fig. 6c and d). Light trapping of long wavelengths (λ > 800 nm) is very important for Si solar cell, which is directly related to improved performance of the solar cell using the low photon-energy effectively^41^,^43^. The nanocone shaped Si device is effective to focus the photons of longer wavelengths and prominently improved QE values for longer wavelengths.

In summary, we have reported the record high efficiency of 16.3% among the nanoscale patterned Si solar cells. Periodically patterned Si nanocones were achieved by a large area applicable nanoimprint method. SiO_2_-masks were arrayed on a planar Si substrate and were effective to provide the periodic Si nanocone by using a wet-etching process. The nanocone-shaped surface is enormously reduces the reflection of incident light and also drives more photons into the Si material. Beside these optical benefits, the nanocone-embedded emitter is electrically advantageous to have graded doping profiles via nano entities. According to the high n-doping concentration, an enhanced electric field is formed through the nanocone structure. This enhanced electric field existing in the nanocone regime is beneficial to collect the photo-generated carriers and also provides a low resistive path. Meanwhile, the nanocone interconnect region has a relatively low dopants, and thus which significantly relieves the recombination loss of photo-generated carrier. We demonstrated the high-performing nanoscale patterned Si solar cell, which realizes the optical benefits into the real electrical improvement. We suggest that this type of nanoscale solar cell will achieve further enhanced performance by controlling the doping profiles of an emitter and scaling of nanostructures for light propagation.

## Methods

### PMMA patterning

Large-scale applicable nanoimprint method applied to nanoscale patterned PMMA layer. A spin coating method was used to deposit 250 nm-thick PMMA layer on a 4-inch Si wafer, at 1000 rpm for 60 s. After then, UV-curable resin was coated onto the PMMA layer. To make nanoscale patterns in the UV-resin, UV imprinting process was performed by using an NIL-8 imprinter (Obducat, Sweden) under exposing 365 nm-UV light (25 mW/cm^2^). O_2_ reactive ion etching (O_2_-RIE) process was followed to tailor the UV-resin-sitting PMMA layer. During this etching process, the nanoscale-patterned UV-resin layer works as an etching mask to transmit its shape to nanoscale hole-arrayed PMMA patterns. A field emission scanning electron microscope (FESEM, FEI Sirion) was employed to observe the Si structures. The doping concentration profile was investigated by secondary ion mass spectroscopy (SIMS, Cameca, magnetic sector ims7f).

### Junction formation

The p/n junction was obtained by a thermal doping method. We used a Czochralski (CZ) grown 4-inch p-type (100) Si wafer, having a doping level of 10^16^/cm^3^. This p-Si wafer loaded in a doping furnace and the n-doping process was performed. During the doping process, a temperature was maintained at 800°C. Phosphoryl oxychloride (POCl_3_) was flown as a doping agency with the mixed N_2_ and O_2_ gases for 30 min. A buffered hydrofluoric acid (5% HF) solution was used to remove phosphosilicate glass (PSG).

### ITO layer coating

In order to deposit ITO layer on a Si substrate, a DC magnetron sputtering system (SNTEK, Korea) was used. During the sputtering, a DC power source (3.70 W/cm^2^) was applied to a 4-inch ITO target (In_2_O_2_ containing 10 wt% SnO_2_). This was performed under Ar atmospheric condition at a temperature of 300°C.

### Electrode formation

The front and back metal electrodes were fabricated for solar cells. A back electrode was achieved by a printing method. Al paste was printed on the back Si substrate and a firing process was performed to establish a back surface field. For a front electrode, a grid pattern was employed by a screen printing of Ag paste on the ITO film, followed by dry process at 150°C for 5 min.

### One-sun simulation

A simulator system (McScience-K3000, Korea) was employed to measure solar cell performances. A photovoltaic power meter (McScience-K101) was used to monitor the I–V characteristics under one sun (100 mW/cm^2^) illumination. Carrier collection efficiencies of solar cells were profiled by using a quantum efficiency measurement system (McScience-K3100).

## Author Contributions

J.K. conceived this research. J.H.Y. designed the selective emitter solar cell devices. H.K. fabricated solar cells. Y.C. performed FDTD simulation. H.H.P. fabricated PMMA and SiO_2_ patterns. M.M.D.K. analyzed device performances. J.Y. and W.A.A. contributed to design solar cells. J.Y. supervised H.K., D.W.K. supervised Y.C. All the authors contributed to prepare this manuscript.

## Supplementary Material

Supplementary Information Supplementary Information

## Figures and Tables

**Table 1:** Device performances

	Planar Si	Texture Si	Nanocone Si
I_RS_ (mA)	0.3	2.4	0.9
I_F_ (A)	3.79	3.58	4.12
Ideality factor	1.52	1.61	1.57
Rectifying ratio	15152.5	1499.5	4367.3
Surface enhancement	100%	N/A	133.9%
V_oc_ (mV)	584	586	583
J_sc_ (mA/cm^2^)	32.05	32.86	36.25
Cell efficiency (%)	14.5	15.0	16.3
