# Supplementary material for: Transparent conductor-embedding nanocones for selective emitters: optical and electrical improvements of Si solar cells
Source: Sci Rep. 2015 Mar 19;5:9256. doi: 10.1038/srep09256 (PMC4365400; doi:10.1038/srep09256)
Supplement: Supplementary Information [file srep09256-s1.pdf]

Supplementary Information

Transparent conductor-embedding nanocones for selective emitters: Optical and electrical improvements of Si solar cells

Joondong Kim,[1]* Ju-Hyung Yun,[1] Hyunyub Kim,[2] Yunae Cho,[3] Hyeong-Ho Park,[4] M. Melvin David Kumar,[1] Junsin Yi,[2] Wayne A. Anderson,[5] and Dong-Wook Kim[3]*

[1]Department of Electrical Engineering, Incheon National University, Incheon 406772, Korea

[2]College of Information and Communication Engineering, Sungkyunkwan University, Suwon 440746, Korea

[3]Department of Physics, Ewha Womans University, Seoul 120750, Korea

[4]Patterning Process Department, Nano Process Division, Korea Advanced Nano Fab Center, Suwon 443270, Korea

[5]Department of Electrical Engineering, University at Buffalo, State University of New York, Buffalo, New York 14260, USA

*Correspondence to: joonkim@incheon.ac.kr (J. Kim) and dwkim@ewha.ac.kr (D.W. Kim)

Calculation of solar cell efficiency

The solar cell efficiency (η) is the ratio of the maximum power output (P_m) over the incident power (P_{in}), which can be calculated by the following equation:

$$\eta = \frac{P_m}{P_{in}} = \frac{V_m J_m}{P_{in}} \quad (1)$$

where, V_m and J_m are the voltage and current density values at P_m point (MPP), which can be found by plotting of power curves of a solar cell. For a nanocone solar cell, we found the values (V_m=0.482 V and J_m=33.804 mA/cm^2), corresponding to the power density of 16.3 mW/cm^2 by using a simulator system (McScience-K3000) under one-sun (100 mW/cm^2) illumination in connection with a power meter (McScience-K101). From equation (1), we obtained the solar cell efficiency of 16.3% for nanocone solar cell.

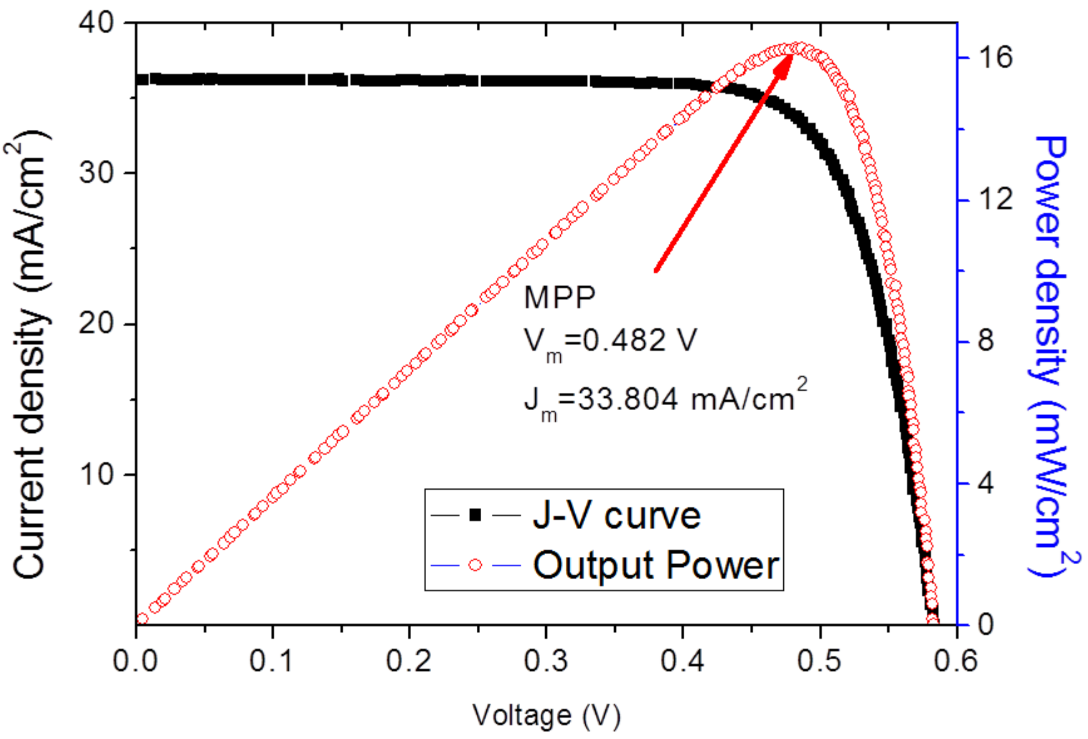

Supplementary Fig. 1| Nanocone Si solar cell power curves. The maximum power output point was marked as MPP and which represent the output efficiency over the incident power.

Relative EQE

To overlook the structural influences over the carrier collection performances, the relative EQE values of textured Si and nanocone Si solar cells were plotted with respect to the planar Si solar cell (Supplementary Fig. 2). As a reference, the EQE value of a planar Si solar cell is considered as 100%.

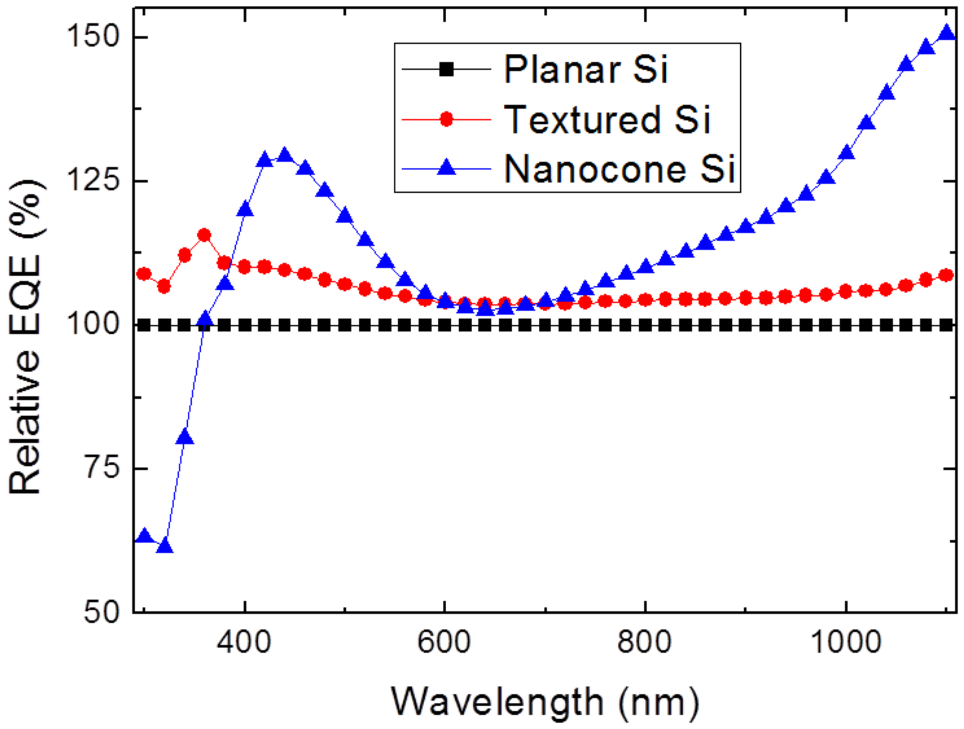

Supplementary Fig. 2| Relative EQE enhancements of the textured Si and the nanocone Si solar cells over a reference value of planar Si.
